# All providers Better Communication Skills (ABCs) program: protocol for a randomized controlled trial assessing communication training effectiveness with interprofessional clinicians

**DOI:** 10.1186/s12904-025-01954-5

**Published:** 2025-11-29

**Authors:** Hsien Seow, Anish K. Arora, Daryl Bainbridge, Zhimeng Jia, Leah Steinberg, Nadia Incardona, Oren Levine, Justin J. Sanders, Jessica Simon, Amanda Roze des Ordons, Karen Zhang, Jeff Myers

**Affiliations:** 1 Department of Oncology, Faculty of Health Sciences, McMaster University, 699 Concession Street Suite 4-204, Hamilton, ON L8V 5C2 Canada; 2 Department of Health Sciences, Faculty of Science, Wilfrid Laurier University, Waterloo, ON Canada; 3 Juravinski Cancer Centre, Hamilton, ON Canada; 4 Department of Family & Community Medicine, Temerty Faculty of Medicine, University of Toronto, Toronto, ON Canada; 5 Department of Family Medicine, Faculty of Medicine & Health Sciences, McGill University, Montreal, QC Canada; 6 Department of Oncology, Cumming School of Medicine, University of Calgary, Calgary, AB Canada; 7 Department of Critical Care Medicine, Cumming School of Medicine, University of Calgary, Calgary, AB Canada; 8 Department of Psychiatry and Behavioural Neurosciences, McMaster University, Hamilton, ON Canada

**Keywords:** Serious illness communication, Health professions education, Medical education, Training programs, Health personnel, Interprofessional, Professional competence, Continuing professional development, Randomized controlled trial

## Abstract

**Background:**

High-quality person-centred communication for those living with serious illness benefits patients, families, and health care professionals (HCPs). However, research suggests that HCPs often find it challenging to engage in these discussions. The purpose of this study is to assess the effectiveness of the ‘All providers Better Communication Skills’ (ABCs), an online blended learning program delivered over three months. The ABCs program seeks to complement existing programs by adopting an interprofessional and person-centred educational philosophy, teaching core communication skills that can be utilized by any clinician across many conversations and healthcare settings, and investigating outcomes focused on clinician’s skill acquisition and behavior change.

**Methods:**

We will conduct a Canada-wide prospective stepped-wedge randomized controlled trial with interprofessional HCPs. All participants will receive the intervention, however, there will be a delay in the intervention for those randomly assigned to the control group. Our study will measure change in pre and post intervention scores for all participants. The primary outcome consists of external expert rater assessments of participants’ video-recorded Standardized Patient (SP) encounters using the validated Assessment of Clinical Encounters – Communication Tool. Secondary outcomes include: participant self-assessments on self-efficacy using the validated Self-Efficacy-12 measure, as well as competence using the Patient and Family-Centered Communication subscale from the validated End-of-Life Professional Caregiver Survey; and SP ratings using the validated Questionnaire on the Quality of Physician-Patient Interaction and Feeling Heard and Understood measures. Learning experience and perceived usability of the program will also be assessed through the validated Blended Learning Usability Evaluation – Questionnaire and semi-structured interviews following program completion.

**Discussion:**

This study is a national trial evaluating the effectiveness of a communication program for interprofessional HCPs. This research will generate evidence on ways of improving conversations about serious illness. With improved communication skills, clinicians can better support patients and families in their illness understanding, deliver care that better aligns with their patients’ goals and values, improve care-related outcomes and experiences, and optimize healthcare resources – all of which can support health system strengthening.

**Trial registration:**

https://clinicaltrials.gov/study/NCT06606470.

**Supplementary Information:**

The online version contains supplementary material available at 10.1186/s12904-025-01954-5.

## Background

The growing body of research on serious illness communication focuses on what, when, and how conversations take place between health care professionals (HCPs) and people living with serious illnesses, their substitute decision makers (SDMs), and their family caregivers [1, 2]. Serious illness communication encompasses a wide variety of topics, such as breaking bad news, code status discussions, and advance care planning. These conversations are important in helping people prepare for and make medical decisions, and should occur throughout a patient’s illness journey, not only near end of life [3].

Effective communication helps patients better understand their illness, and is associated with improved quality of life, higher satisfaction, and an experience of care that is consistent with their priorities [4–6]. Family members also report higher satisfaction with care and improved bereavement outcomes when they have a better understanding of the severity of their loved one’s illness [7, 8]. Unfortunately, many patients and families have a limited understanding of their disease trajectory and severity [8–14], assuming it is curable and/or often greatly overestimating survival [9–11]. While it is important for HCPs to competently facilitate these conversations, evidence shows that they rarely engage patients in conversations about serious illness [15–17]. Research shows many HCPs have not received sufficient communication skills training and feel unprepared to engage in such conversations [18–22].

To explore what programs exist that aim to address this educational need, as well as understand efficacy evidence for existing programs, our team previously conducted a scoping review and two systematic reviews [23–25]. We found that while several serious illness communication training programs exist, they are heterogenous in their intended target audience and topics covered, rely heavily on in-person delivery which limits efforts for large-scale spread, and often lack objective measurement of skill acquisition or behaviour change following program completion [23–25]. The result is conflicting evidence on effectiveness and challenges to wide-spread implementation. To complement existing programs, we developed the ‘All providers Better Communication Skills’ (ABCs) program. The ABCs program focuses on teaching interprofessional HCPs to engage in open, non-judgemental, curious, and empathic communication, with the aim of improving communication throughout serious illness journeys, not only at crisis or decision points.

### Aims

We intend to conduct a randomized controlled trial of the ABCs program. The primary objective of this study is to examine the impact of the training intervention on HCP communication skill acquisition and objective behaviour change, through external expert rater assessments of video recorded standardized patient (SP) encounters. The secondary objectives of this study are to: examine the impact of the training intervention on HCP communication self-efficacy and competency through learner self-assessments; SP perspectives on the quality of learner communication; and learner perceptions on the usability of this blended program.

## Methods

### Description of the ABCs program

The ABCs is a virtual, person-centred, educational program for interprofessional HCPs that was developed by a large interdisciplinary team of clinicians, researchers, and educators with extensive experience in caring for people living with serious illness. The content of this program is based on over two decades of providing communication workshops in palliative care and a review of existing relevant programs (e.g., Vital Talk [26], Heart to Heart [27], Serious Illness Care Program [22], SPIKES protocol [28], PULSES framework [29], CAPACITI [30], and Pallium LEAP [31]), which the ABCs content is intended to complement, not duplicate. A stakeholder panel including clinicians, educators, patients, and family advisors from across Canada served to define and refine the content of the program.

The ABCs program begins by teaching a novel framework called “Preparing or Deciding”, which helps HCPs understand the overall purpose and outcomes of serious illness conversations [3]. This is followed by education around several principles of communication (e.g., self-awareness, complexity, relational exploration), components of conversations (e.g., preparing yourself, exploring illness understanding, and learning to be a reflective practitioner), and fundamental skills (e.g., listening to understand, responding to emotion, and speaking to be understood) which serve as the building blocks to facilitate effective, compassionate, and empathic communication across clinical encounters regardless of health care setting or discipline. The underlying philosophy guiding the ABCs program is that conversations about serious illness are complex, iterative, and relational, and that the core skills taught in this program can be used across any interaction. The ABCs aims to build comfort with uncertainty and the realization that there are often no “fixes” when talking to people living with a serious and life-limiting illness.

**Fig. 1:**
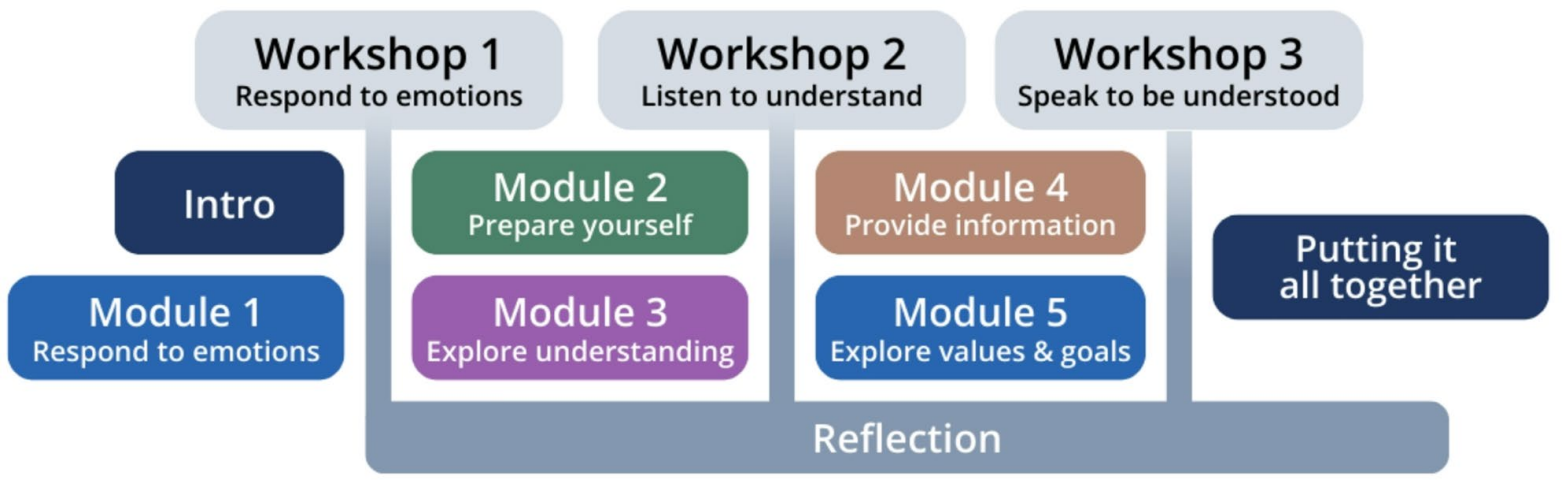
Diagram of ABCs program components

The program adopts a 3-month long online blended learning format (i.e., participants engage in five asynchronous online modules and three synchronous interactive workshops) that provide any clinician (regardless of discipline, specialty, or years in practice) with practical tools, tips, and strategies to implement when communicating with seriously ill patients and their families. We piloted this education program with medical learners and interdisciplinary HCPs [32, 33] and found that completion of the ABCs program improved their self-reported confidence in initiating conversations around serious illness, as well as competency in serious illness communication, as evaluated by external raters. The ABCs program will be accessed via abcseducation.ca, a custom Moodle learning management system which is hosted, developed, and supported by Lingel Learning (i.e., a Moodle certified partner and service provider). Figure 1 shows the program components and organization. Table 1 further describes each of these components.

**Table 1:** The ABC program’s educational strategies and components

Conceptual Framework
• Use of a conceptual framework that clarifies serious illness communication concepts. • Use of a framework that draws from strengths of existing programs but focuses on specific skills that are relevant to learners and clinicians at all training and practice levels, as well as across all disciplines, specialties, and care settings. • Use of a framework grounded in person-centeredness and competency.
Modules
• Asynchronous videos to support development of micro-skills, featuring example encounters with SPs and experts.
Workshops
• Online synchronous sessions featuring case scenarios and simulated encounters with SPs to put concepts into practice and refine communication skills.
Coaching
• Opportunities for experts to observe communication and provide advice through simulated practice.
Personal reflection
• Reflection encouraged following each module and workshop.
Learning management system (LMS)
• A custom Moodle-based LMS that allows participants to easily access online materials, book their SP encounter sessions, and complete study surveys.
Duration
• A three-month long training program which provides sufficient time to gain knowledge, practice skills, reflect on learning, and potentially change behaviour in real clinical contexts.

### Study design

**Fig. 2:**
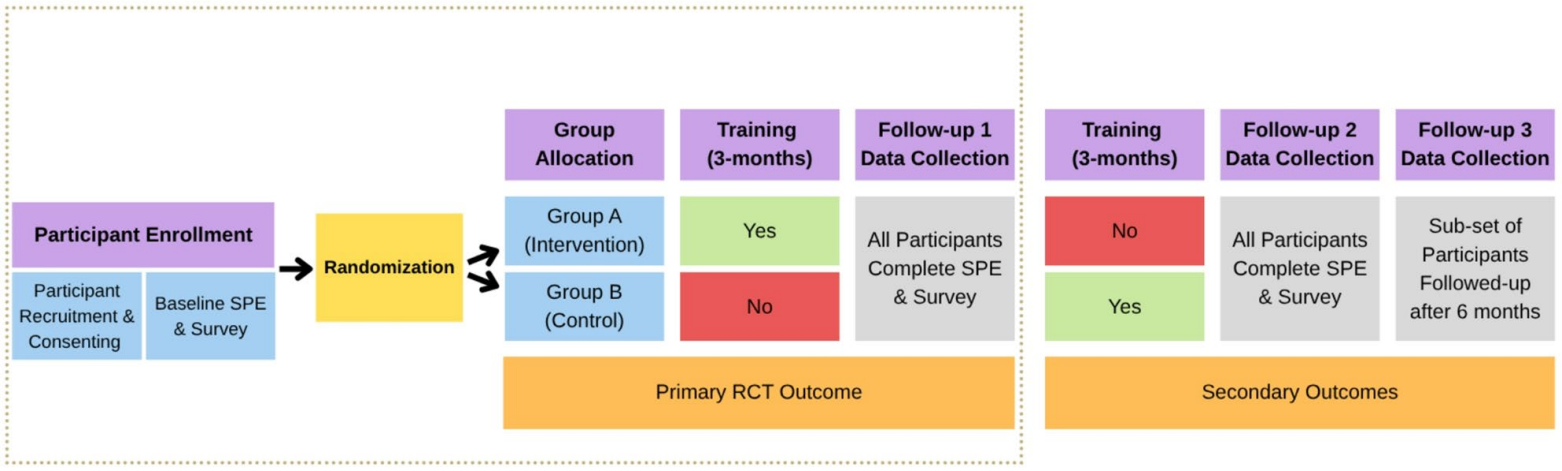
Diagram of randomized controlled trial design

We will conduct a prospective stepped-wedge randomized controlled trial [34] of interprofessional health care providers (HCPs) who enroll in the ABCs program. This stepped-wedge trial will consist of up to three waves of enrolment (first wave in 2025; second in 2027; and a third in 2029). At the start of each wave, all participants that meet eligibility criteria will complete baseline measures. Following this, participants will be randomized to an intervention or a control group. The intervention group will have immediate access to the ABCs program, whereas the control group will have to wait until the intervention group completes the program before they can receive the training. Once the intervention group completes the program, and before the control group begins the program, all study participants will complete follow-up assessments (Follow-up 1). Assessments at Follow-up 1 will serve as the primary outcome measures for this study. All study participants will complete another round of follow-up assessments once the second group completes the program (Follow-up 2). A subset of the participants will also complete a final follow-up 6 months after the cross-over group completes their training (Follow-up 3). Assessments at Follow-ups 2 and 3 will serve as secondary outcome measures for this study. See Fig. 2 for a depiction of the study design’s first wave, which will be repeated at the second and third waves. Ethical approval for this study was obtained from the Hamilton Integrated Research Ethics Board (#17801). See https://clinicaltrials.gov/study/NCT06606470 for the approved operating protocol. The SPIRIT-Outcomes 2022 checklist was corroborated when drafting this protocol (see supplementary material).

### Randomization

Randomization will be managed by a senior research coordinator (DB). HCPs who register for the program during the recruitment period and complete the baseline measures (including the baseline SP encounter) will be randomized to either the intervention or control arm using a computer-generated 1:1 sequence of allocation prior to the first iteration of the ABCs program in this trial. Each assignment will be placed in a spreadsheet which will reveal the allocation of the individual once they complete the baseline measures and are considered enrolled in the ABCs program. Individuals may be stratified by province and/or health profession.

### Study population & eligibility

All interprofessional HCPs who provide direct care to patients are eligible to enroll and participate in the study. HCPs from across Canada will be invited so that our data have geographically diverse representation (e.g. rural, urban, and remote), thereby building evidence on generalizability within diverse communities across the country. To enroll in the ABCs program and study, potential participants need to provide informed consent, as well as complete the program registration form, an online survey, and a simulated encounter with a SP.

### Recruitment

Potential participants will be informed about ABCs through an invitation distributed by our partner groups and organizations (e.g., Choosing Wisely Canada, the Canadian Society of Palliative Care Physicians, Canadian Hospice Palliative Care Association, Hospice Palliative Care Ontario, Unity Health Network, Hamilton Health Sciences, and university departments of all co-investigators and collaborators). In advance of enrollment for each of the study waves, two information webinars will be offered for individuals wanting to learn more about the program and associated research study. Potential participants wishing to enroll in the ABCs program and research will complete the online registration form (using LimeSurvey [35]), in which they will have the opportunity to review, download, and sign the study informed consent form.

The ABCs program will be offered free of charge, with the understanding that those participating will complete the educational and data collection components. While completion of an initial SP encounter and baseline survey is necessary for enrolment into the study and will thus not be compensated, participants will receive gift cards for their completion of each of the follow-up SP encounters ($25 per encounter) and surveys ($25 per survey). Select participants who complete the program will be invited to an interview and/or a six-month follow-up SP encounter and survey ($25 gift card provided for this subsequent activity).

### Evaluative framework

Street et al.’s framework [36] was used to guide development of the ABC program and will also inform its evaluation. This framework links clinician-patient communication with health outcomes, proposing that aspects of “communication functions” (e.g., managing uncertainty, fostering relationships, responding to emotions) can both directly and indirectly impact health outcomes (e.g., less suffering, emotional well-being, and pain control). Indirect pathways can involve proximal outcomes (e.g., increased prognosis understanding) and/or intermediate outcomes (e.g., increased trust in system). The communication functions within the framework were used to define the learning outcomes for the ABCs program (e.g., ensure HCPs can appropriately respond to emotions upon program completion). Participant self-assessments, SP assessments, and expert rater assessments will serve as proximal outcomes. Future studies will be conducted to assess the impact of the ABCs on intermediate and overall health outcomes among patients in clinical settings.

### Outcomes

We will use quantitative (i.e., validated questionnaires) and qualitative (i.e., semi-structured interviews) methods. Quantitative outcomes will be measured for all participants at three timepoints: baseline, upon program completion by the group that receives the intervention immediately, and upon program completion by the late start group. Quantitative and qualitative outcomes for select participants will also be collected after 6-months post program completion by all participants. Table 2 describes the study outcome measures and scoring methods.

**Table 2:** Outcome measures and scoring

Outcomes	Response options/Sub-domains	Scoring
**External Rater Assessment Measure**		
• Assessment of Clinical Encounter-Communications Tool (ACE-CT)	14 items, 5-point Likert scale 5 Sub-domains: Speaks to be Understood Listens to Understand Responds to Emotion Conversational Flow Closing the Conversation Scored on a 5-point scale ranging from: 1. Intervention needed 2. Direction needed 3. Support needed 4. Competence demonstrated 5. Mastery demonstrated 6. Not assessable in this conversation	Individual item scores (mean of each) Sub-domain and overall summary scores = average of all items
**Participant Self-Assessment Measures**		
• Self-Efficacy Questionnaire (SE-12)	12 items, 10-point Likert scale ranging from of 1 (Very uncertain) to 10 (Very certain) or Not relevant	Individual item scores (mean of each) Overall summary score = sum of all items
• End-of-Life Professional Caregiver Survey (EPCS) [Patient and Family-Centered Communication sub-domain only]	12 items, 5-point Likert scale Patient and Family-Centered Communication (PFCC 12-items) EPCS Items 1 to 12 Scored on a 5-point scale ranging from 1 (lowest level of skill) to 5 (greatest level of skill). 1 = Not at all 2 = A little bit 3 = Somewhat 4 = Quite a bit 5 = Very Much 99 = Don’t Know or Not Applicable	Individual item scores (mean of each) Overall summary score (PFCC) = sum of all items
• Self-assessment of impact of and satisfaction with the intervention: Blended Learning Usability Evaluation – Questionnaire (BLUE-Q) Note: Post Intervention Only	32 items, 5-point Likert scale 4 Parts: Course Content Synchronous Learning Environment Asynchronous Learning Environment Overall Impressions of the Course Scored on a 5-point scale ranging from 1 (Strongly disagree) to 5 (Strongly agree) 1 = Strongly disagree 2 = Disagree 3 = Neutral 4 = Agree 5 = Strongly agree	Individual item scores (mean of each)
**Standardized Patient Assessment Measures**		
• Questionnaire on Quality of Physician-Patient Interaction (QQPPI) (select items)	14 items (7 items selected), 5-point Likert scale ranging from 1 (do not agree) to 5 (fully agree) 1 = I do not agree 2 = I partly agree 3 = I agree 4 = I strongly agree 5 = I fully agree 99 = Not Applicable	Individual item scores (mean of each) Overall summary score = sum of all items
• Feeling Heard and Understood scale (select items)	10 items (7 items selected), 5-point Likert scale ranging from 1 (Not at all true) to 5 (Completely true) 1 = Not at all true 2 = A little bit true 3 = Somewhat true 4 = Very true 5 = Completely true 99 = Not Applicable	Individual item scores (mean of each) Overall summary score = sum of all items
• Global Assessment of conversation (Overall rating of participant’s communication skills)	1 item, 5-point Likert scale ranging from 1 (Poor) to 5 (Excellent) 1 = Poor 2 = Fair 3 = Good 4 = Very Good 5 = Excellent	Mean score

#### Primary outcomes

The primary outcome for this study is the validated Assessment of Clinical Encounter-Communications Tool (ACE-CT) which external expert raters will used to evaluate the quality of participant communication with SPs in the virtual simulated encounters (Table 2) [37]. The ACE-CT is comprised of 14 items and was developed through a rigorous 3-phase multi-methods process. Through our pilot study, psychometric properties of the ACE-CT were assessed, indicating that the tool has high internal consistency (Cronbach’s ⍺=0.96), moderate inter-rater and intra-rater reliability, and sound content and construct validity.

#### Secondary outcomes

Secondary outcome measures include tools completed by participants for self-assessments and those completed by SPs with whom the participants have simulated encounters (Table 2). We will also report study metrics, including recruitment, randomization, representativeness, adherence to intervention, and completeness of data collection. The main secondary outcomes are as follows:

- Participant (HCP) self-reported measures
  - Self-Efficacy Questionnaire (SE-12) [38]: The SE-12 is a 12-item survey developed to measure the impact of communication skills training from the perspective of participants. The SE-12 has been validated and widely used in different settings and with different HCPs. The SE-12 has demonstrated internal consistency (Cronbach’s α = 0.95) and sufficient test-retest reliability (intraclass correlation agreement = 0.71).
  - End-of-life Professional Caregiver Survey (EPCS) [39]: The EPCS is a 28-item scale developed to assess palliative care-specific educational needs within interprofessional teams, related to three main subdomains: Effective Care Delivery (8-items); Patient and Family-Centered Communication (12-items); and Cultural and Ethical Values (8-items). For the purposes of this study, we only include the Patient and Family-Centered Communication sub-domain. The EPCS exhibits strong internal consistency (alpha = 0.96).
  - Blended Learning Usability Evaluation – Questionnaire (BLUE-Q) (Post intervention only) [40, 41]: The BLUE-Q is a 29-item, three-part questionnaire that was developed and validated by members of this research team. The purpose of the BLUE-Q is to evaluate the usability (i.e., perceived effectiveness, efficiency, satisfaction, accessibility, organization, and overall learning experiences) of blended learning programs, specifically those deployed in the field of health professions education. The BLUE-Q has a total of 23 Likert scale items and 6 open-ended questions. The three parts of the BLUE-Q are: (1) pedagogical usability (e.g., the program content, learning objectives, and experience of learners with their instructors); (2) synchronous learning aspects of the program (e.g., face-to-face workshops); and (3) asynchronous learning aspects of the program (i.e., online learning modules and learning management system).

- Standardized patient perspectives
  - Questionnaire on Quality of Physician-Patient Interaction (QQPPI) [42]: The QQPPI is a 14 item, validated scale. This questionnaire is completed by patients to rate their perceptions on the quality of physician–patient interactions during outpatient care. The QQPPI demonstrates high internal consistency (Cronbach’s α = 0.95) and significant correlations with other quality-related measures.
  - Feeling Heard and Understood scale [43]: This scale has 4 items and is a patient-reported quality measure for palliative care settings. The survey in intended for seriously ill patients to indicate the extent to which they felt heard and understood by their care provider, have established trust with this individual, and that they perceive their care to have a whole-person orientation. The scale has been tested rigorously and found to exhibit sound validity and reliability.

- Qualitative Interviews
  - Semi-structured individual interviews will be conducted via Zoom videoconferencing software with select participants. The interviews will explore longer-term impacts of the ABCs education program, applicability to practice, behavior changes, potential patient or family impacts, challenges to implementation, and learning context.

### Statistical power/sample size

The primary analysis will be a linear regression analysis, where the outcome variable is the ACE-CT score at Follow-up 1, and covariates are the baseline ACE-CT score, stratification variables, and intervention arm. If the intervention arm covariate is statistically significant, then the intervention will be deemed as statistically significant. However, for accurate sample size estimation for such a regression analysis, one would need not only the hypothesized change in ACE-CT scores from pre to post intervention, but also the estimated correlation for all the stratification factors and the distribution of personnel across factors. As these estimates are unlikely to be accurate, a simplified sample size estimate can be generated by assuming a two-sample t-test and adding an inflation factor. Hence, using data from our pilot study [37], it is estimated that the pre-intervention global mean for ACE-CT is 2.90 (standard deviation [SD] = 0.73), the post-intervention global mean for ACE-CT is 3.45 (SD = 0.78) and the mean change from pre- to post-intervention is 0.54 (SD = 0.75) amongst individuals undergoing the ABCs program. It is assumed there will be no change in the post-intervention global mean for ACE-CT amongst individuals who do not receive the program. Based on these assumptions, a two-sided, two-sample equal variance t-test (alpha = 0.05) will achieve 90% power with 42 HCPs per arm (84 total). To account for the different analytic method use, potential overestimation of the hypothesized effect, and to account for potential non-completion by an estimated 20% of HCPs, the target sample size will be doubled to 168.

### Data collection

Data sources will consist of (1) participant surveys, (2) SP surveys, (3) external rater assessments of SP encounter video recordings, and (4) virtual interviews with participants. All surveys will be completed on-line through SurveyMonkey [44] for SPs and external raters, and the Moodle learning management system for participants. All virtual workshop activities will be hosted using the Zoom Video communications platform [45] and coordinated through the research team.

Participant and SP completed measures will be collected at four time points: at baseline (i.e., prior to intervention), upon intervention group completion of the program (within 8 weeks after completion), upon control group completion of the intervention (within 8 weeks after completion), and at six months following the intervention. Participants will be asked to sign up for a virtual SP encounter at each data collection point. The initial SP encounter must be completed before the participant gains access to the ABCs curriculum on Moodle. The next module in sequence will only be released after completion of the previous module. Each SP encounter will occur online through Zoom and will be video recorded. A clinical scenario will be provided in advance, and a SP will simulate the encounter. The same clinical scenario will be used for all simulated encounters. Virtual encounters will be capped at 20 min. No immediate feedback will be provided to participants.

The video recordings will be reviewed and rated asynchronously by trained raters. These raters are interdisciplinary HCPs (e.g., physicians, nurses, psychologists, social workers) who are trained to provide education in serious illness communication to clinician learners. Raters will also be trained to use the rating scale (ACE-CT), given a rating manual, and will be engaged in a virtual calibration session. Raters will be blinded to the training condition (control/intervention conditions) and to the data collection time-point. Raters are not involved in the delivery of the interactive workshops. All video recorded encounters from a participant will be independently assessed by a randomly assigned rater, with 15% duplication between raters to test for consistency in ratings.

Select participants will be asked to participate in qualitative data collection at Follow up 3. Purposive sampling will be used to ensure a diversity in participants and perspectives. We will complete qualitative interviews with 15 to 25 selected participants, or until thematic saturation is reached. All identifying information will be removed from data once collected, with use of unique coded identifiers to maintain participant anonymity. All study data will be password-protected and maintained on McMaster University servers.

### Data analysis

The intervention and unit of analysis will be at the individual level. Descriptive statistics (i.e., means, standard deviations, and frequencies) will summarize recruitment, population demographics, and adherence to study procedures. Participant characteristics (e.g., province, prior training, etc.) will be tabulated. Regression analyses will be conducted to analyze relationships between independent variables and outcomes, and to compare group means across different levels of categorical variables. Multilevel mixed models with repeated measures will also be used to investigate the effect of the intervention over time (baseline, follow-up 1, 2, and 3). Correlations between participant, SP, and rater responses to the respective outcome scales will be performed using Pearson’s coefficient. Interview audio files will be transcribed verbatim and reviewed for accuracy. Thematic analysis guided by Braun and Clarke’s framework [46] will be conducted independently by two researchers to identify key aspects of participants’ experiences, with core themes developed through group discussion. NVIVO software will be used for qualitative data management.

## Discussion

Serious illness communication skills provide a foundation for high quality care among those who are at risk of progressive illness and dying. Yet, most HCPs do not feel comfortable or confident in such conversations, and this impacts patients’ and their family’s healthcare outcomes and experiences. Through this study, we will rigorously evaluate the ABCs program, which focuses on teaching basic communication skills and principles that can be applied across any discipline, care setting, and throughout illness trajectories. Moreover, we hypothesize that an online blended learning approach, coupled with simulated practice with SPs, expert feedback, and opportunities for guided self-reflection throughout the program will facilitate long-term behaviour change. The online blended approach can support learners in better controlling their learning experience (e.g., pacing and timing of their education) [47–51], whereas the other program elements may give learners more time to reflect on and practice the skills taught [52, 53].

This study has several methodological strengths. In this study, all participants eventually receive the intervention, however, delayed access to the program for those randomized to the late group still enable robust comparisons between intervention and control groups. Additionally, evaluating the effectiveness of the program from various perspectives (i.e., learner self-assessments, SP assessments, external expert rater assessments) and through the use of multiple validated measures will provide a comprehensive evaluation. The addition of qualitative interviews will further strengthen the study by helping us better understand why certain outcomes were observed, how the program was received, and how it could be improved for future iterations. Including participants from diverse geographic and professional backgrounds will increase the generalizability of findings and enhance the potential for widespread implementation.

While the study design ensures that all participants eventually receive the intervention, staggered access to the intervention may introduce variability in external factors (e.g., seasonal variance, political or economic changes, exposure to other communication training), which may influence participation or outcomes. Participant attrition may occur particularly among the control group who must wait until the intervention group completes the program before being able to begin learning. However, in our prior pilot studies of the ABCs program, the dropout rate was only 9 to 20% [32, 33]. Also, potential attrition has been accounted for in the sample size calculation. Another limitation is that participants that engage in this study may already be motivated to learn and improve their serious illness communication skills, creating a potential selection bias. Also, the assessment of SP encounters through video recordings, while valuable, may not fully capture the complexity of real-world patient interactions.

## Conclusions

This study is among the first nation-wide randomized controlled trials to evaluate the impact of a virtual communication training program for interprofessional HCPs in Canada. We uniquely use a range of measures, including the validated ACE-CT scored by independent raters, to measure skill acquisition in clinician learners. By leveraging an online blended model with asynchronous modules alongside synchronous SP encounters, workshops, and coaching, delivered over a 3-month period, the ABCs program has the potential to enhance serious illness communication skills across diverse healthcare settings. Findings from this study will provide critical insights into the effectiveness of the intervention. If effectiveness in skill development is demonstrated, the ABCs program could serve as a scalable model for improving communication among HCPs, ultimately contributing to improvements in patient-centred care, higher quality of life and satisfaction among those living with serious illness, and better experiences for patients, their families, and the HCPs who care for them.

## Supplementary Information

Supplementary Material 1

## Data Availability

The datasets used and/or analysed during the current study are available from the corresponding author on reasonable request.

## Abbreviations

ABCs: All providers Better Communication Skills
HCPs: Health Care Providers
SPs: Standardized Patients
